# Nine Genes Mediate the Therapeutic Effects of Iodine-131 Radiotherapy in Thyroid Carcinoma Patients

**DOI:** 10.1155/2020/9369341

**Published:** 2020-06-16

**Authors:** Han Shuwen, Yang Xi, Da Miao, Xu Jiamin, Zhuang Jing, Gao Weili

**Affiliations:** ^1^Department of Oncology, Huzhou Cent Hosp, Affiliated Cent Hosp Huzhou University, 198 Hongqi Rd, Huzhou, Zhejiang, China; ^2^Department of Oncology, Huzhou Central Hospital, 198 Hongqi Road, Huzhou, Zhejiang Province 313000, China; ^3^Department of Nursing, Huzhou Third Municipal Hospital, No. 2088 East Tiaoxi Road, Huzhou, Zhejiang Province 313000, China; ^4^Graduate School of Nursing, Huzhou University, No. 1 Bachelor Road, Huzhou, Zhejiang Province 313000, China; ^5^Department of Thyroid Surgery, Huzhou Central Hospital, No. 198 Hongqi Road, Huzhou, Zhejiang Province 313000, China

## Abstract

**Background:**

Thyroid carcinoma (THCA) is one of the most common malignancies of the endocrine system, which is usually treated by surgery combined with iodine-131 (I^131^) radiotherapy.

**Aims:**

This study is aimed at exploring the potential targets of I^131^ radiotherapy in THCA.

**Methods:**

The RNA-sequencing data of THCA in The Cancer Genome Atlas database (including 568 THCA samples) was downloaded. The differentially expressed genes (DEGs) between the tumour samples whether or not subjected to I^131^ radiotherapy were identified using edgeR package. Using the WGCNA package, the module that was relevant with I^131^ radiotherapy was selected. The intersection genes of the hub module nodes and the DEGs were obtained as hub genes, followed by the function and pathway enrichment analyses using the clusterProfiler package. Moreover, the protein-protein interaction (PPI) network for the hub genes was constructed using Cytoscape software. In addition, more important hub genes were analysed with function mining using the GenCLiP2 online tool. The qPCR analysis was used to verify the mRNA expression of more important hub genes in THCA tissues.

**Results:**

There were 500 DEGs (167 upregulated and 333 downregulated) between the two groups. WGCNA analysis showed that the green module (428 nodes) exhibited the most significant correlation with I^131^ radiotherapy. A PPI network was built after the identification of 53 hub genes. In the PPI network, *CDH5*, *KDR*, *CD34*, *FLT4*, *EMCN*, *FLT1*, *ROBO4*, *PTPRB*, and *CD93* exhibited higher degrees, which were mainly implicated in the vascular function. The relative expression of nine mRNAs in the THCA tissues treated with I^131^ was lower.

**Conclusion:**

I^131^ radiotherapy might exert therapeutic effects by targeting *CDH5*, *KDR*, *CD34*, *FLT4*, *EMCN*, *FLT1*, *ROBO4*, *PTPRB*, and *CD93* in THCA patients.

## 1. Introduction

As one of the most common malignancies of the endocrine system, thyroid carcinoma (THCA) is classified into papillary (PTC), follicular (FTC), medullary (MTC), and undifferentiated types [1]. Although THCA only accounts for 2% of all malignant tumours, it has attracted attention due to a younger target population and higher incidence [2]. In particular, anaplastic THCA usually has a poor outcome and is responsible for 14–39% of THCA-related deaths [3, 4].

There have been several guidelines for the diagnosis and initial disease management for the treatment of thyroid carcinoma [5]. The most recent American Thyroid Association guidelines (2009) recommend total thyroidectomy for tumours > 1 cm and possible lobectomy for tumours ≤ 1 cm [6]. Nevertheless, when THCA invades a relatively wider area, the tumours sometimes cannot be completely removed surgically [7]. Therefore, some patients need to be treated with iodine-131 (I^131^) radiotherapy postoperatively to destroy any remaining neoplastic cells that were not removed during surgery [8–10]. I^131^ is a radioactive isotope of iodine, which can kill human tissue cells [11]. To avoid environmental and human contamination, radioiodine treatment usually uses an I^131^ dose of 100–200 mCi with the patient in total isolation for 48 h. This therapy has shown positive results in terms of reducing recurrence rates in patients with well-differentiated THCA [12]. However, the molecular mechanisms underlying the effects of I^131^-related radiotherapy on THCA remain poorly understood.

In this study, we intended to explore the key genes that respond to I^131^ radiotherapy. RNA-sequencing data of THCA patients whether or not subjected to I^131^ radiotherapy were downloaded from a public database, followed by a series of bioinformatics analyses. The qPCR analysis was used to verify the mRNA expression of hub genes in THCA tissues.

## 2. Materials and Methods

### 2.1. Data Source

The University of California Santa Cruz's (UCSC's) Xena platform (http://xena.ucsc.edu/) contains the public data from databases, such as The Cancer Genome Atlas (TCGA, https://cancergenome.nih.gov/), International Cancer Genome Consortium (http://www.icgc.org), and Therapeutically Applicable Research to Generate Effective Treatments (http://target.cancer.gov). The data on this platform was standardized for subsequent analysis [13]. The RNA-sequencing data and relevant clinical data of THCA in the TCGA database (including 568 THCA samples and 615 phenotypes) were downloaded using the UCSC Xena platform [13].

### 2.2. Data Preprocessing

Using the R package biomaRt (version 2.38.0, http://bioconductor.org/packages/biomaRt/) [14], the serial numbers of genes were converted into gene symbols. The serial numbers that could not be matched to gene symbols were deleted. When multiple serial numbers corresponded to the same gene symbol, the average value of the serial numbers was taken as the final expression value of this gene symbol and was used for subsequent analysis.

The clinical expression matrices that matched to the gene symbols were extracted according to gene types, and only mRNAs were extracted for subsequent analysis. The mRNAs with low expression were difficult to be verified and could interfere with the results of differential expression analysis, and thus, these mRNAs (expressing in at least 25% samples) were removed. The remaining genes were included in the subsequent analysis.

Normal samples were removed from the clinical expression profile of THCA. From the remaining tumour samples, the samples with or without clear clinical information of receiving I^131^ radiotherapy were screened and the samples without such records were deleted. The eligible tumour samples were used for the subsequent analysis.

### 2.3. Differential Expression Analysis

To analyse the gene expression differences between the tumour samples whether or not subjected to I^131^ radiotherapy, differential expression analysis was conducted using the R package, edgeR (version 3.24.3, http://bioconductor.org/packages/edgeR/) [15]. The threshold for screening differentially expressed genes (DEGs) was set at *p* value ≤ 0.01.

### 2.4. Weighted Gene Coexpression Network Analysis (WGCNA)

WGCNA is an algorithm that can be used to cluster genes with similar expression patterns and construct a gene coexpression network [16]. First, the WGCNA algorithm assumed that the gene network obeys a scale-free distribution and defined the correlation matrix of the gene coexpression and the adjacency function formed by the gene network. Then, the dissimilarity coefficient of different nodes was calculated, and the hierarchical clustering tree was constructed accordingly. Finally, the associations between modules and specific phenotypes or diseases were explored, and the target genes and gene networks for disease treatment were ultimately identified. Using the R package, WGCNA (version 1.66, https://cran.r-project.org/web/packages/WGCNA/) [16], WGCNA analysis was carried out. In detail, the expression matrix obtained through data preprocessing was subjected to log_2_(*x* + 1) standardization, and the genes with median absolute deviation in the top 75% and larger than 0.01 were screened. After screening and removing the outlier, the one-step method was used to build the WGCNA network, and each module was visualized in the hierarchical clustering tree. Finally, receiving I^131^ treatment or not was used as the external biological parameter for performing correlation analysis of the modules, and the module that was the most highly correlated with the external biological parameter was selected for further study.

### 2.5. Functional and Pathway Enrichment Analyses

After the module that was most highly correlated to I^131^ treatment was identified, the module nodes were subjected to Gene Ontology (GO) [17] function and Kyoto Encyclopedia of Genes and Genomes (KEGG) [18] pathway enrichment analyses using the clusterProfiler package (version 3.10.1, http://bioconductor.org/packages/clusterProfiler/) [19] in R. The thresholds for screening the enrichment results were defined at adjusted *p* value < 0.01 and *q* value < 0.05.

Using Cytoscape software (http://www.cytoscape.org/) [20], the key module was analysed and the degree of each module node was calculated. The module nodes with degree > 200 were selected as hub nodes. By comparing the hub nodes with the DEGs, the intersection genes that were statistically significant and played key roles in the module were identified as hub genes. Furthermore, enrichment analysis for the hub genes was conducted using the clusterProfiler package [19]. The screening threshold was set at adjusted *p* value < 0.05.

### 2.6. Protein-Protein Interaction (PPI) Network Analysis

Using the STRING database (http://string-db.org/) [21], PPI analysis for the hub genes was conducted. The threshold for PPI analysis used the default values in the database. The obtained PPI pairs were visualized in the PPI network using Cytoscape software [20], and the network nodes with degree > 5 were selected as more important hub genes.

### 2.7. Function Mining of More Important Hub Genes

Using the GenCLiP2 online tool (http://ci.smu.edu.cn/GenCLiP2/analysis.php) [22], the functions of the more important hub genes were analysed through literature mining. The enrichment results with *p* value < 1*e*-4 and involving at least four genes were selected.

### 2.8. Collection of Tissue Samples

The study protocol was approved by the Medical Ethics Committee of Huzhou Central Hospital (China). Informed consent was obtained from patients or guardians. In total, 47 THCA patients subjected to I^131^ radiotherapy and 50 THCA patients without preoperative therapy at the Huzhou Central Hospital from March 2018 to March 2019 were included in the present study. The pathological diagnosis of THCAs was confirmed by two senior pathologists. The collected tissue samples were snap-frozen and stored at -80°C.

### 2.9. qPCR Analysis

A TRIzol reagent (Invitrogen, Carlsbad, USA) was used to isolate the total RNA. The First-Strand cDNA Synthesis kit (K1622; Thermo Fisher, USA) was used to synthesize the first-strand cDNA. Quantitative real-time RT-PCR was performed using a PCR reaction mixture (2 *μ*l primer mixture, 10 *μ*l SYBR Green, 7 *μ*l DEPC water, and 1 *μ*l diluted cDNA). The transcripts were detected using real-time fluorescence quantitative PCR (ABI StepOnePlus Real-Time PCR system, Applied Biosciences, USA). The thermocycling conditions were as follows: 95°C for 5 min, followed by 40 cycles of 95°C for 15 sec, 60°C for 20 sec, and 72°C for 40 sec. The experiments were repeated three times. The sequences of the primers were shown in supplemental material Table S1. The GAPDH was as the internal control, and the lowest Ct value of gene expression in TACH patients was as the baseline. The 2^-*ΔΔ*Ct^ method was used to calculate the mRNA expression, and the results were expressed as fold changes.

## 3. Results

### 3.1. Data Preprocessing and Differential Expression Analysis

**Figure 1:**
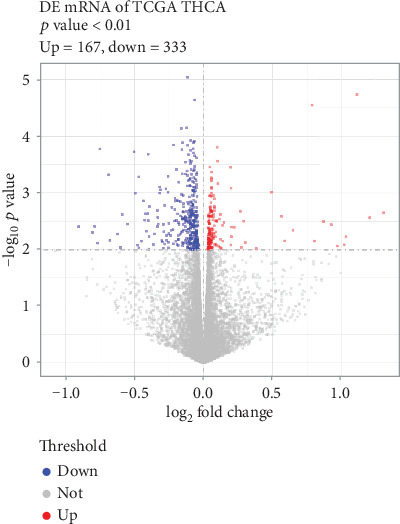
The DEGs between the THCA samples whether or not subjected to I^131^ radiotherapy. The RNA-sequencing data and relevant clinical data of THCA in the TCGA database were used to screen the DEGs between the THCA samples whether or not subjected to I^131^ radiotherapy. The volcano plot shows the differentially expressed genes (DEGs) between the THCA samples whether or not subjected to I^131^ radiotherapy. Each dot represents a gene. Red, blue, and grey dots represent upregulated genes, downregulated genes, and non-DEGs, respectively. TCGA: The Cancer Genome Atlas; THCA: thyroid carcinoma.

After data preprocessing, a total of 17003 genes and 298 tumour samples were selected. Therefore, a 17003 × 298 expression matrix was obtained for the subsequent analysis. There were 500 DEGs between the tumour samples whether or not subjected to I^131^ radiotherapy, including 167 upregulated genes and 333 downregulated genes. The volcano plot of the DEGs is presented in Figure 1.

### 3.2. WGCNA Analysis

**Figure 2:**
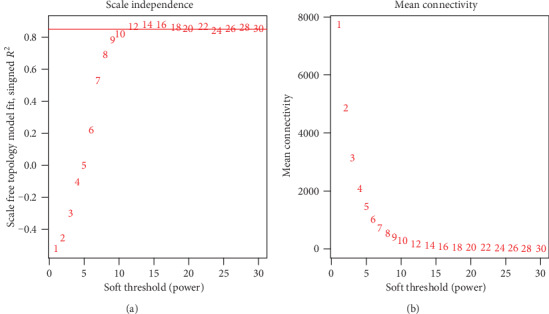
Selection of soft threshold in WGCNA. WGCNA is an algorithm that can be used to cluster genes with similar expression patterns and construct a gene coexpression network. The selection of a soft threshold is a very important task for WGCNA. The selection principle of a soft threshold is to make the constructed network accord with the characteristics of a scale-free network. The figure shows the calculation results of a soft threshold (power) in WGCNA. The two indicators including the scale-free topology model fit and mean connectivity tend to be flat, indicating that the selected soft threshold is appropriate. (a) The selection graph of a soft threshold. (b) The mean connectivity of genes under different soft thresholds.

After preprocessing, a 12751 × 298 matrix was obtained for WGCNA analysis. In the process of outlier screening, one outlier was found in all samples. After removing the outlier, the 12751 × 297 matrix was used for constructing the WGCNA network. In WGCNA analysis, the soft threshold (power) was selected as 12 (Figure 2(a)). The mean connectivity tended to zero as the soft threshold got larger, indicating that the calculation of the soft threshold was reliable (Figure 2(b)).

**Figure 3:**
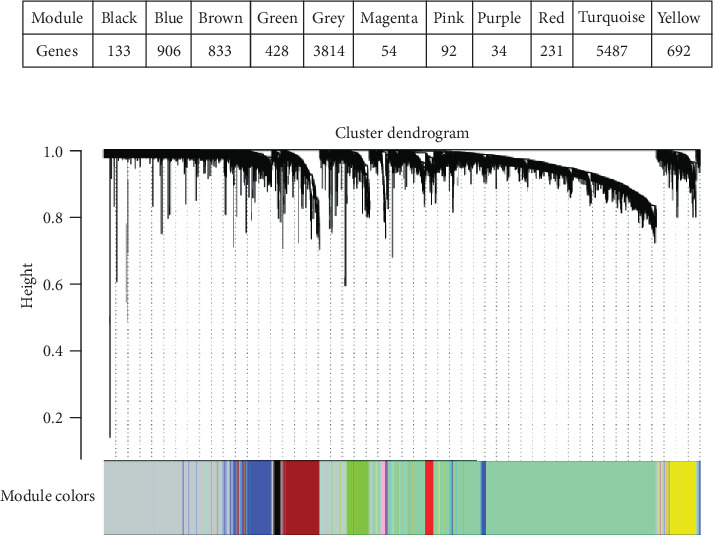
The hierarchical clustering tree of WGCNA. The module that was relevant with I^131^ radiotherapy in THCA was analysed by using the WGCNA. The expression matrix obtained through data preprocessing was subjected to log_2_(*x* + 1) standardization, and the genes with median absolute deviation in the top 75% and larger than 0.01 were screened. After screening and removing the outlier, the one-step method was used to build the WGCNA network, and each module was visualized in the hierarchical clustering tree. The table showed the number of clustered genes in the functional modules. The figure showed the hierarchical clustering tree of the weighted gene coexpression network. Grey indicates a cluster of genes that are not included in any of the functional modules.

The weighted gene coexpression network was built and visualized in the hierarchical clustering tree. There were a total of 10 functional modules (black module, 133 nodes; blue module, 906 nodes; brown module, 833 nodes; green module, 428 nodes; magenta module, 54 nodes; pink module, 92 nodes; purple module, 34 nodes; red module, 231 nodes; turquoise module, 5487 nodes; and yellow module, 692 nodes) (Figure 3).

**Figure 4:**
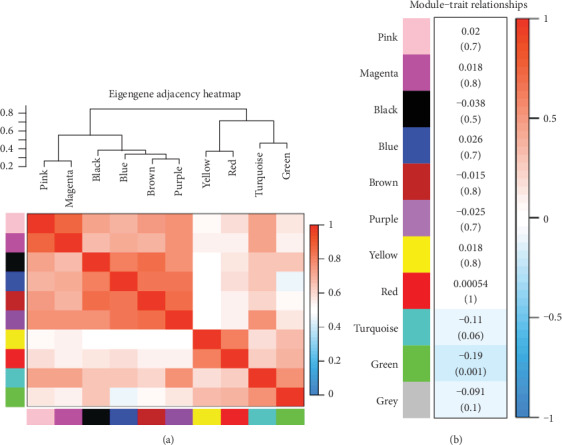
Correlation analysis among the functional modules and module-trait relationships. The figure showed a correlation analysis among the functional modules and module-trait relationships. (a) The correlations among the functional modules (the modules divided into the same branch have similar functions). (b) The correlations between each functional module and phenotype (whether or not subjected to I^131^ radiotherapy).

**Figure 5:**
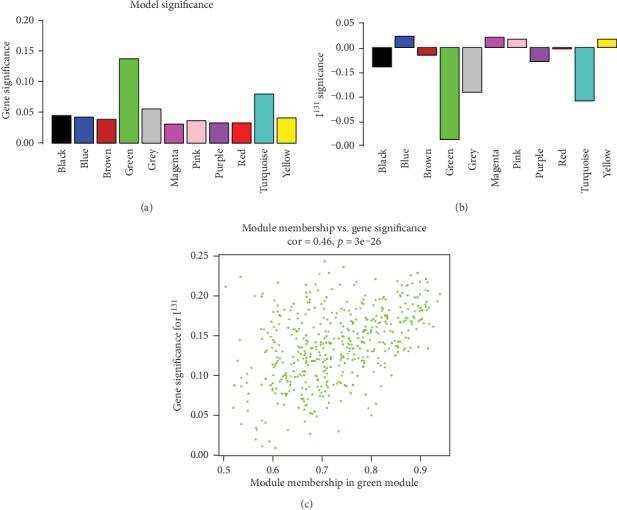
The correlation analysis of the significant functional module for THCA patients subjected to I^131^ radiotherapy. The confirmation of green module and the correlation analysis for green module. (a) The correlation between each functional module and gene. (b) The correlation between I^131^ radiotherapy and gene. (c) The results of the correlation analysis for the green module.

Both correlation analysis among the functional modules (Figure 4(a)) and correlation analysis between each functional module and phenotype (whether or not subjected to I^131^ radiotherapy) (Figure 4(b)) were conducted. Our results showed that the green module exhibited the most significant correlation with I^131^ radiotherapy. The genes in the green module were listed in the supplementary material (Table S2). Therefore, the green module was selected for the subsequent analysis. In addition, the correlation between each functional module and gene (Figure 5(a)) and the correlation between I^131^ radiotherapy and gene (Figure 5(b)) were separately analysed. The genes involved in the green module had the strongest correlations with all genes. These results suggested that the green module was the most relevant and significant functional module for THCA patients subjected to I^131^ radiotherapy. The results of the correlation analysis indicated that the genes in the green module were significantly correlated not only with the corresponding module but also with I^131^ radiotherapy (Figure 5(c)). Thus, the genes in the green module deserved further exploration.

### 3.3. Functional and Pathway Enrichment Analyses

To explore the biological functions and pathways involving the components of the green module, the 428 genes in the green module were subjected to enrichment analysis. The enriched functions mainly included circulatory system processes (GO biological progress (BP), adjusted *p* value = 1.72*E*-19, and *q* value = 6.23*E*-16), extracellular matrix (GO cellular component (CC), adjusted *p* value = 1.20*E*-09, and *q* value = 4.34*E*-07), and channel activity (GO molecular function (MF), adjusted *p* value = 4.03*E*-07, and *q* value = 1.24*E*-04). In addition, the pathway enrichment analysis showed that the 428 genes were mainly involved in focal adhesion (adjusted *p* value = 7.10*E*-08 and *q* value = 1.14*E*-05), cGMP-PKG signaling pathway (adjusted *p* value = 1.04*E*-07 and *q* value = 1.14*E*-05), and Rap1 signaling pathway (adjusted *p* value = 5.92*E*-07 and *q* value = 3.33*E*-05) (Table 1).

A total of 70 hub nodes with a degree > 200 were identified from the green module and were then compared with the 500 DEGs. Finally, 53 intersection genes were obtained as hub genes. These hub genes were implicated in 15 GO biological progress (BP) functional terms (such as endothelium development, adjusted *p* value = 1.69*E*-08), two GO cellular component (CC) functional terms (such as cell-cell junction, adjusted *p* value = 6.60*E*-03), four GO molecular function (MF) functional terms (such as transmembrane receptor protein kinase activity, adjusted *p* value = 2.65*E*-04), and three KEGG pathways (such as focal adhesion, adjusted *p* value = 2.06*E*-03) (Table 2).

### 3.4. PPI Network Analysis

**Figure 6:**
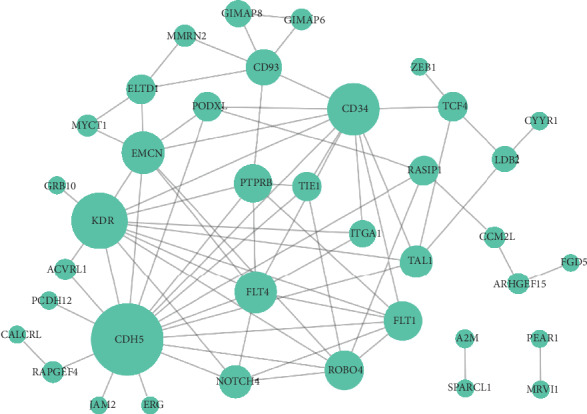
The protein-protein interaction (PPI) network for the hub genes. The larger the node, the greater the degree of the node is.

A PPI network for the hub genes was constructed, which included 38 nodes and 73 edges (Figure 6). With the degree > 5, cadherin 5 (*CDH5*, degree = 17), kinase insert domain receptor (*KDR*, degree = 12), cluster of differentiation antigen 34 (*CD34*, degree = 11), fms-related tyrosine kinase 4 (*FLT4*, degree = 8), endomucin (*EMCN*, degree = 8), *FLT1* (degree = 7), roundabout guidance receptor 4 (*ROBO4*, degree = 7), protein tyrosine phosphatase receptor type B (*PTPRB*, degree = 6), and *CD93* (degree = 6) in the PPI network were selected as more important hub genes.

### 3.5. Function Mining of More Important Hub Genes

**Figure 7:**
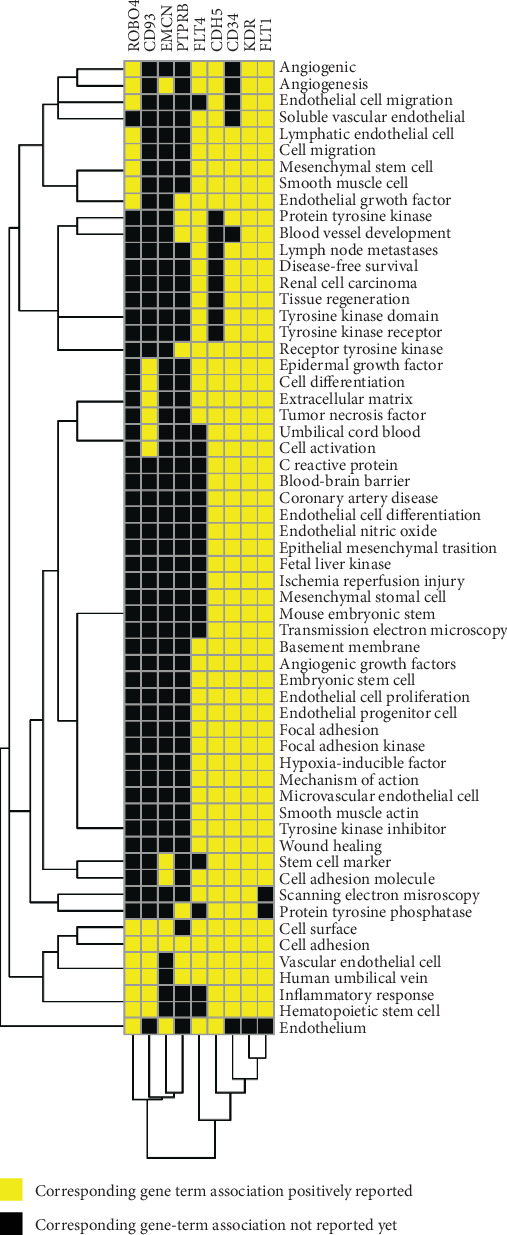
The gene function heatmap based on literature mining. The figure shows the gene function heatmap based on literature mining. Yellow and black represent corresponding gene term association positively reported and corresponding gene term association not reported yet, respectively.

The functions of the nine more important hub genes were mined from the PubMed database. The enrichment analyses showed that these more important hub genes were mainly related to vascular function (such as angiogenesis, cell adhesion, cell activation, and necrosis) (Figure 7).

### 3.6. qPCR Analysis of Hub Genes in Tissues

**Figure 8:**
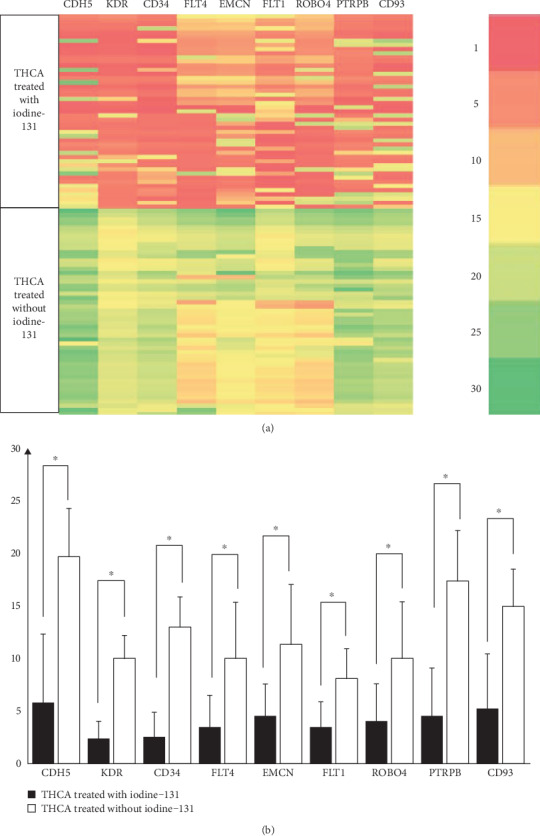
The relative mRNA expression of the hub genes. The heatmap (a) showed the relative mRNA expression of nine genes including CDH5, KDR, CD34, FLT4, EMCN, FLT1, ROBO4, PTPRB, and CD93 in THCA tissues that are treated with or without I^131^. The color scale at the top right represents the relative expression of mRNA. The histogram (b) showed that the relative expression of nine mRNAs in the THCA treated with I^131^ group was lower than that in the THCA treated without I^131^ group, and the differences were statistically significant (∗ represents *p* < 0.05, independent sample *t-*test).

The nine more important hub genes were verified in the clinical tissue samples. The relative mRNA expression of nine hub genes in THCA tissues was shown in Figure 8. The experimental group and control group were the 47 THCA samples treated with I^131^ and 50 THCA samples not treated with I^131^, respectively. The mRNAs of the nine genes including CDH5, KDR, CD34, FLT4, EMCN, FLT1, ROBO4, PTPRB, and CD93 showed a lower expression compared to the control group (*p* < 0.05, independent sample *t-*test).

## 4. Discussion

Our study, for the first time, investigated the key genes that respond to I^131^ radiotherapy in THCA by analysis of the RNA-sequencing data. A total of 500 DEGs (167 upregulated and 333 downregulated genes) between the tumour samples whether or not subjected to I^131^ radiotherapy were obtained. Among the 10 functional modules identified by WGCNA, the green module was the most relevant and significant functional module for THCA patients receiving I^131^ radiotherapy. After comparing the 70 hub nodes in the green module with the 500 DEGs, 53 intersection genes were selected as hub genes. For the 53 hub genes, 15 GO BP functional terms, two GO CC functional terms, four GO MF functional terms, and three KEGG pathways were enriched. From the PPI network, *CDH5*, *KDR*, *CD34*, *FLT4*, *EMCN*, *FLT1*, *ROBO4*, *PTPRB*, and *CD93* were selected as more important hub genes, which were mainly implicated in the vascular function. Vascular functions are the basis of normal metabolism of tumour cells [23]. Thus, I^131^ radiotherapy might affect the normal metabolism of tumour cells and kill tumour cells by influencing their vascular function.

Snail and N-cadherin exhibit inducible and constitutive expression in PTC and may affect the development and progression of PTC. Therefore, Snail and N-cadherin may serve as effective markers for the tumour [24]. N-cadherin contributes to thyroid tumourigenesis by regulating epithelial-mesenchymal transition and certain signaling pathways and may be considered a promising therapeutic target for THCA [25]. The plasma membrane protein, ROBO4, serves as a therapeutic target in tumour endothelial cells; ROBO4 conjugates suppress tumour angiogenesis and growth [26, 27]. Protein tyrosine phosphatase receptor type J (*PTPRJ*) has been reported to be a tumour susceptibility gene and acts as a tumour suppressor gene during the carcinogenesis of THCA [28]. These findings suggested that *CDH5*, *ROBO4*, and *PTPRB* might be associated with the mechanisms of THCA. Thus, they may serve as target genes of I^131^ radiotherapy.

The enrichment analysis for the 428 genes in the green module shows that the hub genes are associated with the vasculature of tumour tissue but not the tumour cells themselves (Table 1). Tumour microenvironment may be involved in the regulation of the iodine-131 radiotherapy in THCH. For example, the HIF1a or VHL-HIF1a axis is targeted by iodine therapy of THCH. The iodine target in tumour cells is HIF1a which could lead to microvessel decrease. Therefore, further studies to evaluate the relationship between the hub genes and HIF1a or VHL-HIF1a axis may provide new research directions for iodine-131 radiotherapy of THCH.

Vascular endothelial growth factor (*VEGF*), vascular endothelial growth factor receptor 1 (*VEGFR1*, also called *FLT1*), and *VEGFR2* (also known as *KDR*) are upregulated in PTC, and inhibiting *VEGFR* offers a novel approach for managing the development of THCA [29]. Both *VEGFR2* and RET protooncogenes in MTC are targeted by the superior dual inhibitor, AGN-PC-0CUK9P, and thus, AGN-PC-0CUK9P may be used for improving the therapy of MTC [30]. The activities of genes in tumour vessels (such as *VEGFR2* and *VEGFR3* (also called *FLT4*)) and tumour cells can be suppressed by the multikinase inhibitor, sorafenib, and sorafenib exerts antiangiogenic and antiproliferative effects in advanced THCA [31]. Through a cell-mediated approach, soluble FLT1 has been found to be able to repress tumour angiogenesis and growth in patients with FTC [32]. Therefore, I^131^ might also function in THCA treatment by targeting *KDR*, *FLT4*, and *FLT1*.

*CD34* is implicated in cell adhesion and signal transduction and is an indicator of neoplastic behaviour, and CD34^+^ stromal cells are related to the carcinogenesis of PTC [33]. Precursor CD34^+^ stromal cells or monocytes can develop into dendritic cells (DCs), and a higher number of DCs may contribute to the favourable prognosis of PTC [34]. The transmembrane receptor, CD93, is highly expressed in tumour vessels of multiple cancers, indicating that CD93 functions by organizing the extracellular matrix and mediating vascular maturation [35, 36]. The expression of five genes (*EMCN*, engulfment and cell motility 1, solute carrier organic anion transporter family member 2A1, potassium voltage-gated channel subfamily A member regulatory beta subunit 1, and inter-alpha-trypsin inhibitor heavy chain 5) decreased in FTC compared with benign follicular thyroid adenoma (FTA), and the 5-gene classifier has high specificity and sensitivity and can be used to distinguish FTC from FTA [37]. Taken together, the differential expressions of *CD34*, *CD93*, and *EMCN* after I^131^ radiotherapy indicated that the three genes may be targets of I^131^ radiotherapy.

Although nine key genes were identified to be associated with I^131^ radiotherapy in THCA, these genes were not validated in multicenter and large samples, which was a limitation of this study. Whereas I^131^ is radioactive and cell experiment is difficult to be carried out, the cell experiments could not be performed. In the follow-up clinical treatment, the weight of genes would be comprehensively considered and a gene screening model based on the nine genes may be constructed to provide guidance for the selection of therapeutic options.

In conclusion, *CDH5*, *KDR*, *CD34*, *FLT4*, *EMCN*, *FLT1*, *ROBO4*, *PTPRB*, and *CD93* might respond to I^131^ radiotherapy of THCA patients. In addition, I^131^ radiotherapy might kill THCA cells by affecting vascular functions.

## Figures and Tables

**Table 1:** The results of enrichment analysis for the 428 genes in the green module (top 10 listed).

Category	Description	Count	*p* adjust	*q* value
Biological process	GO:0003013~circulatory system process	49	1.72*E* − 19	6.23*E* − 16
	GO:0008015~blood circulation	48	5.00*E* − 19	9.07*E* − 16
	GO:0043062~extracellular structure organization	39	1.52*E* − 15	1.84*E* − 12
	GO:1901342~regulation of vasculature development	37	1.84*E* − 13	1.67*E* − 10
	GO:0030198~extracellular matrix organization	33	4.85*E* − 13	3.52*E* − 10
	GO:0032835~glomerulus development	15	1.16*E* − 12	6.23*E* − 10
	GO:0045765~regulation of angiogenesis	34	1.20*E* − 12	6.23*E* − 10
	GO:0002040~sprouting angiogenesis	23	1.20*E* − 11	5.45*E* − 09
	GO:0001570~vasculogenesis	15	5.55*E* − 11	2.24*E* − 08
	GO:0001667~ameboidal-type cell migration	34	1.67*E* − 10	6.07*E* − 08
Cell component	GO:0031012~extracellular matrix	34	1.20*E* − 09	4.34*E* − 07
	GO:0062023~collagen-containing extracellular matrix	25	4.13*E* − 08	7.50*E* − 06
	GO:0043235~receptor complex	27	9.19*E* − 08	1.11*E* − 05
	GO:0043292~contractile fiber	18	1.80*E* − 06	1.64*E* − 04
	GO:0044449~contractile fiber part	17	3.02*E* − 06	2.18*E* − 04
	GO:0005604~basement membrane	11	3.60*E* − 06	2.18*E* − 04
	GO:0045177~apical part of cell	23	4.58*E* − 06	2.37*E* − 04
	GO:1902495~transmembrane transporter complex	21	5.82*E* − 06	2.64*E* − 04
	GO:0098857~membrane microdomain	20	7.80*E* − 06	3.07*E* − 04
	GO:1990351~transporter complex	21	8.45*E* − 06	3.07*E* − 04

Molecular function	GO:0015267~channel activity	29	4.03*E* − 07	1.24*E* − 04
	GO:0022803~passive transmembrane transporter activity	29	4.21*E* − 07	1.24*E* − 04
	GO:0022838~substrate-specific channel activity	27	1.40*E* − 06	2.75*E* − 04
	GO:0005216~ion channel activity	26	2.34*E* − 06	3.44*E* − 04
	GO:0008528~G protein-coupled peptide receptor activity	14	3.30*E* − 06	3.88*E* − 04
	GO:0001653~peptide receptor activity	14	5.32*E* − 06	5.21*E* − 04
	GO:0005201~extracellular matrix structural constituent	14	7.21*E* − 06	0.000606
	GO:0019838~growth factor binding	13	8.94*E* − 06	6.57*E* − 04
	GO:0004115~3′,5′-cyclic-AMP phosphodiesterase activity	5	1.17*E* − 05	7.63*E* − 04
	GO:0071813~lipoprotein particle binding	6	2.50*E* − 05	1.34*E* − 03

Pathway	hsa04510~focal adhesion	20	7.10*E* − 08	1.14*E* − 05
	hsa04022~cGMP-PKG signaling pathway	18	1.04*E* − 07	1.14*E* − 05
	hsa04015~Rap1 signaling pathway	19	5.92*E* − 07	3.33*E* − 05
	hsa04512~ECM-receptor interaction	12	6.10*E* − 07	3.33*E* − 05
	hsa04151~PI3K-Akt signaling pathway	25	1.52*E* − 06	6.61*E* − 05
	hsa05032~morphine addiction	12	1.92*E* − 06	6.97*E* − 05
	hsa04270~vascular smooth muscle contraction	14	3.78*E* − 06	01.18*E* − 04
	hsa04020~calcium signaling pathway	16	1.35*E* − 05	3.67*E* − 04
	hsa04611~platelet activation	12	4.78*E* − 05	1.15*E* − 03
	hsa05165~human papillomavirus infection	21	5.31*E* − 05	1.15*E* − 03

GO: Gene Ontology.

**Table 2:** The Gene Ontology (GO) functional terms and pathways enriched for the 53 hub genes.

Category	Description	Count	*p* adjust
Biological process	GO:0003158~endothelium development	7	1.69*E* − 08
	GO:0001570~vasculogenesis	6	2.66*E* − 08
	GO:0048010~vascular endothelial growth factor receptor signaling pathway	6	7.30*E* − 08
	GO:0030947~regulation of vascular endothelial growth factor receptor signaling pathway	4	1.08*E* − 06
	GO:0045602~negative regulation of endothelial cell differentiation	3	2.72*E* − 06
	GO:0045601~regulation of endothelial cell differentiation	4	3.21*E* − 06
	GO:0045446~endothelial cell differentiation	5	6.41*E* − 06
	GO:0061298~retina vasculature development in camera-type eye	3	1.00*E* − 05
	GO:0045765~regulation of angiogenesis	7	2.96*E* − 05
	GO:0090287~regulation of cellular response to growth factor stimulus	6	5.17*E* − 05
	GO:1901342~regulation of vasculature development	7	5.58*E* − 05
	GO:0001935~endothelial cell proliferation	5	8.52*E* − 05
	GO:0045766~positive regulation of angiogenesis	5	1.08*E* − 04
	GO:0031589~cell-substrate adhesion	6	1.40*E* − 04
	GO:0038084~vascular endothelial growth factor signaling pathway	3	1.45*E* − 04

Cell component	GO:0043235~receptor complex	8	7.12*E* − 04
	GO:0005911~cell-cell junction	7	6.60*E* − 03

Molecular function	GO:0019199~transmembrane receptor protein kinase activity	5	2.65*E* − 04
	GO:0004714~transmembrane receptor protein tyrosine kinase activity	4	1.15*E* − 03
	GO:0019838~growth factor binding	5	1.15*E* − 03
	GO:0070888~E-box binding	3	9.27*E* − 03

Pathway	hsa04510~focal adhesion	4	2.06*E* − 03
	hsa04015~Rap1 signaling pathway	4	2.34*E* − 03
	hsa04670~leukocyte transendothelial migration	3	3.64*E* − 03

## Data Availability

The data used to support the findings of this study are available from the corresponding author upon request.

## Abbreviations

THCA: Thyroid carcinoma
I^131^: Iodine-131
DEGs: Differentially expressed genes
PPI: Protein-protein interaction
PTC: Papillary thyroid carcinoma
FTC: Follicular thyroid carcinoma
MTC: Medullary thyroid carcinoma
UCSC: University of California Santa Cruz
TCGA: The Cancer Genome Atlas
WGCNA: Weighted Gene Coexpression Network Analysis
GO: Gene Ontology
KEGG: Kyoto Encyclopedia of Genes and Genomes
BP: Biological progress
CC: Cellular component
MF: Molecular function
CDH5: Cadherin 5
KDR: Kinase insert domain receptor
CD34: Cluster of differentiation antigen 34
FLT4: fms-related tyrosine kinase 4
EMCN: Endomucin
ROBO4: Roundabout guidance receptor 4
PTPRB: Protein tyrosine phosphatase receptor type B
VEGF: Vascular endothelial growth factor
VEGFR1: Vascular endothelial growth factor receptor 1.

## Supplementary Materials

Supplementary 1 Table S1: the primers of hub genes

Supplementary 2 Table S2: nodes—green.

## References

[1] Pan J. J., Zhao L., Cheng R., Yang Y., Hu Y. H. (2017). Thyroid carcinoma in children and adolescents: clinical characteristics and follow-up from two centers. *Journal of Cancer Research and Therapeutics*.

[2] Sanabria A., Kowalski L. P., Shah J. P. (2018). Growing incidence of thyroid carcinoma in recent years: factors underlying overdiagnosis. *Head & Neck*.

[3] Baloch Z. W., Livolsi V. A. (2018). Special types of thyroid carcinoma. *Histopathology*.

[4] Adeniran A. J., Chhieng D. (2016). Anaplastic thyroid carcinoma. *Head & Neck*.

[5] Sherma S. I. (2003). Thyroid carcinoma. *The Lancet*.

[6] Smallridge R. C., Ain K. B., Asa S. L. (2012). American Thyroid Association guidelines for management of patients with anaplastic thyroid cancer. *Thyroid*.

[7] Albano D., Bertagna F., Panarotto M. B., Giubbini R. (2017). Early and late adverse effects of radioiodine for pediatric differentiated thyroid cancer. *Pediatric Blood & Cancer*.

[8] Hjiyej L. A., Aschawa H., Mellouki I., Anwar W. (2019). Iodine-131 therapy for thyroid cancers: estimation of radiation doses received by relatives and contact restriction times. *Radioprotection*.

[9] Salvatori M., Luster M. (2010). Radioiodine therapy dosimetry in benign thyroid disease and differentiated thyroid carcinoma. *European Journal of Nuclear Medicine and Molecular Imaging*.

[10] Middendorp M., Grünwald F. (2010). Update on recent developments in the therapy of differentiated thyroid cancer. *Seminars in Nuclear Medicine*.

[11] Wang Y., Liu C., Wang J., Zhang Y., Chen L. (2017). Iodine-131 induces apoptosis in human cardiac muscle cells through the p53/Bax/caspase-3 and PIDD/caspase-2/t-BID/cytochrome c/caspase-3 signaling pathway. *Oncology Reports*.

[12] Patel Supriya S., Melanie G. (2013). Well-differentiated thyroid carcinoma: the role of post-operative radioactive iodine administration. *Journal of Surgical Oncology*.

[13] Casper J., Zweig A. S., Villarreal C. (2018). The UCSC genome browser database: 2018 update. *Nucleic Acids Research*.

[14] Zhang J., Haider S., Baran J. (2011). BioMart: a data federation framework for large collaborative projects. *Database*.

[15] Nikolayeva O., Robinson M. D. (2014). *edgeR* for differential RNA-seq and ChIP-seq analysis: an application to stem cell biology. *Stem Cell Transcriptional Networks*.

[16] Tang X., Huang X., Wang D. (2019). Identifying gene modules of thyroid cancer associated with pathological stage by weighted gene co-expression network analysis. *Gene*.

[17] Zhao Y., Fu G., Wang J., Guo M., Yu G. (2019). Gene function prediction based on Gene Ontology Hierarchy Preserving Hashing. *Genomics*.

[18] Yang D., He Y., Wu B. (2020). Integrated bioinformatics analysis for the screening of hub genes and therapeutic drugs in ovarian cancer. *Journal of Ovarian Research*.

[19] Yu G., Wang L. G., Han Y., He Q. Y. (2012). clusterProfiler: an R package for comparing biological themes among gene clusters. *OMICS*.

[20] Mustafin Z. S., Lashin S. A., Matushkin Y. G., Gunbin K. V., Afonnikov D. A. (2017). Orthoscape: a cytoscape application for grouping and visualization KEGG based gene networks by taxonomy and homology principles. *BMC Bioinformatics*.

[21] Szklarczyk D., Morris J. H., Cook H. (2017). The STRING database in 2017: quality-controlled protein-protein association networks, made broadly accessible. *Nucleic Acids Research*.

[22] Lin H., Ma Y., Wei Y., Shang H. (2016). Genome-wide analysis of aberrant gene expression and methylation profiles reveals susceptibility genes and underlying mechanism of cervical cancer. *European Journal of Obstetrics, Gynecology, and Reproductive Biology*.

[23] Cantelmo A. R., Pircher A., Kalucka J., Carmeliet P. (2017). Vessel pruning or healing: endothelial metabolism as a novel target?. *Expert Opinion on Therapeutic Targets*.

[24] Yang X., Shi R., Zhang J. (2016). Co-expression and clinical utility of Snail and N-cadherin in papillary thyroid carcinoma. *Tumour Biology*.

[25] Da C., Wu K., Yue C. (2017). N-cadherin promotes thyroid tumorigenesis through modulating major signaling pathways. *Oncotarget*.

[26] Dai C., Gong Q., Cheng Y., Su G. (2019). Regulatory mechanisms of Robo4 and their effects on angiogenesis. *Bioscience Reports*.

[27] Zhao H., Ahirwar D. K., Oghumu S. (2016). Endothelial Robo4 suppresses breast cancer growth and metastasis through regulation of tumor angiogenesis. *Molecular Oncology*.

[28] Iuliano R., Palmieri D., He H. (2010). Role of PTPRJ genotype in papillary thyroid carcinoma risk. *Endocrine-Related Cancer*.

[29] Razy N. H. M. P., Rahman W. F. W. A., Win T. T. (2019). Expression of vascular endothelial growth factor and its receptors in thyroid nodular hyperplasia and papillary thyroid carcinoma: a tertiary health care centre based study. *Asian Pacific Journal of Cancer Prevention*.

[30] Dunna N. R., Kandula V., Girdhar A. (2015). High affinity pharmacological profiling of dual inhibitors targeting RET and VEGFR2 in inhibition of kinase and angiogeneis events in medullary thyroid carcinoma. *Asian Pacific Journal of Cancer Prevention*.

[31] Corrado A., Ferrari S. M., Politti U. (2017). Aggressive thyroid cancer: targeted therapy with sorafenib. *Minerva Endocrinologica*.

[32] Ye C., Feng C., Wang S. (2004). sFlt-1 gene therapy of follicular thyroid carcinoma. *Endocrinology*.

[33] Daoud S. A., Esmail R. S. E. N., Hareedy A. A., Khalil A. (2015). Stromal modulation and its role in the diagnosis of papillary patterned thyroid lesions. *Asian Pacific Journal of Cancer Prevention*.

[34] Batistatou A., Zolota V., Scopa C. D. (2002). S-100 protein+ dendritic cells and CD34+ dendritic interstitial cells in thyroid lesions. *Endocrine Pathology*.

[35] Lugano R., Vemuri K., Yu D. (2018). CD93 promotes *β*1 integrin activation and fibronectin fibrillogenesis during tumor angiogenesis. *Journal of Clinical Investigation*.

[36] Greenlee-Wacker M. C., Galvan M. D., Bohlson S. S. (2012). CD93: recent advances and implications in disease. *Current Drug Targets*.

[37] Pfeifer A., Wojtas B., Oczko-Wojciechowska M. (2013). Molecular differential diagnosis of follicular thyroid carcinoma and adenoma based on gene expression profiling by using formalin-fixed paraffin-embedded tissues. *BMC Medical Genomics*.

