# Supplementary material for: Nine Genes Mediate the Therapeutic Effects of Iodine-131 Radiotherapy in Thyroid Carcinoma Patients
Source: Dis Markers. 2020 Jun 16;2020:9369341. doi: 10.1155/2020/9369341 (PMC7317313; doi:10.1155/2020/9369341)
Supplement: Supplementary 1 — Table S1: the primers of hub genes [file 9369341.f1.docx]

Table S1. The primers of hub genes

| Genes | primers |
| --- | --- |
| CDH5 | F: 5’-TCACCTTCTGCGAGGATATGG-3’  R:5’-GAGTTGAGCACCGACACATC-3’ |
| KDR | F:5’-CGCCAA ATATTTTGGGAAATAGCGGGAAAG -3’  R:5’- TTGTTTGGCCAGTATAATTGTAGTTTAAAACG -3’ |
| CD34 | F: 5’-TGAAAAAGCTGGGGATCCTAGA-3’  R: 5’-TCCCAGGTCCTGAGCTATAGCC-3’ |
| FLT4 | F:5’-AGCTGGGACCACAGTGGCTGA-3’  R:5’-GGAGGCAGGGGCCAAGAAGAT-3’ |
| EMCN | F: 5’-TCACACCAACAACTGGAACAA-3’  R: 5’-TCAGTGGTTGTGGCTTTCAA-3’ |
| FLT1 | F:5’-TCCCTTATGATGCCAGCAAGT-3’  R: 5’-CCAAAAGCCCCTCTTCCAA-3’ |
| ROBO4 | F: 5'-AGCCAACTGGACTGTAGTTG-3'  R: 5'-GAGCCATAAAAAGTGCTGGTG-3' |
| PTPRB | F: 5’-GCAGACAGAAAGTGAGCCATGGTCGAG-3’  R: 5’-TCTCAATGCCTTGAATAGACTGGATC-3’ |
| CD93 | F:5’-AGCAAGCCGACACATGAAGA-3’  R:5’-CCACCACCGTCCCCAG-3’ |
| GAPDH | F:5’-TCAGCCGCATCTTCTTTTG-3’  R:5’-GACTCCGACCTTCACCTTC-3’ |
